# Prolonged Primary Rhinovirus Infection of Human Nasal Epithelial Cells Diminishes the Viral Load of Secondary Influenza H3N2 Infection via the Antiviral State Mediated by RIG-I and Interferon-Stimulated Genes

**DOI:** 10.3390/cells12081152

**Published:** 2023-04-13

**Authors:** Hsiao Hui Ong, Jing Liu, Yukei Oo, Mark Thong, De Yun Wang, Vincent T. Chow

**Affiliations:** 1 Department of Otolaryngology, Yong Loo Lin School of Medicine, National University of Singapore, Singapore 117545, Singapore; 2 Infectious Diseases Translational Research Program, Yong Loo Lin School of Medicine, National University of Singapore, Singapore 117545, Singapore; 3 Department of Otolaryngology—Head & Neck Surgery, National University Health System, Singapore 119228, Singapore; 4 Department of Microbiology and Immunology, Yong Loo Lin School of Medicine, National University of Singapore, Singapore 117545, Singapore

**Keywords:** human nasal epithelium, rhinovirus persistence, secondary influenza virus infection, rhinovirus re-infection, innate immune responses, RIG-I, interferon-stimulated genes, MX1, IFITM1

## Abstract

Our previous study revealed that prolonged human rhinovirus (HRV) infection rapidly induces antiviral interferons (IFNs) and chemokines during the acute stage of infection. It also showed that expression levels of RIG-I and interferon-stimulated genes (ISGs) were sustained in tandem with the persistent expression of HRV RNA and HRV proteins at the late stage of the 14-day infection period. Some studies have explored the protective effects of initial acute HRV infection on secondary influenza A virus (IAV) infection. However, the susceptibility of human nasal epithelial cells (hNECs) to re-infection by the same HRV serotype, and to secondary IAV infection following prolonged primary HRV infection, has not been studied in detail. Therefore, the aim of this study was to investigate the effects and underlying mechanisms of HRV persistence on the susceptibility of hNECs against HRV re-infection and secondary IAV infection. We analyzed the viral replication and innate immune responses of hNECs infected with the same HRV serotype A16 and IAV H3N2 at 14 days after initial HRV-A16 infection. Prolonged primary HRV infection significantly diminished the IAV load of secondary H3N2 infection, but not the HRV load of HRV-A16 re-infection. The reduced IAV load of secondary H3N2 infection may be explained by increased baseline expression levels of RIG-I and ISGs, specifically MX1 and IFITM1, which are induced by prolonged primary HRV infection. As is congruent with this finding, in those cells that received early and multi-dose pre-treatment with Rupintrivir (HRV 3C protease inhibitor) prior to secondary IAV infection, the reduction in IAV load was abolished compared to the group without pre-treatment with Rupintrivir. In conclusion, the antiviral state induced from prolonged primary HRV infection mediated by RIG-I and ISGs (including MX1 and IFITM1) can confer a protective innate immune defense mechanism against secondary influenza infection.

## 1. Introduction

In clinical settings, it is not uncommon to detect infections with multiple viruses in patients. Repeated human rhinovirus (HRV) infections are common, and several episodes can occur in the same individual within a year due to the large number of serotypes and the type-specific immunity [1,2,3]. Multiple HRV re-infections, in conjunction with other respiratory viruses (such as RSV), are also reported in infants aged 5 to 7 months with underlying diseases, who subsequently progress to recurrent refractory wheezing [4]. HRV infections or re-infections in early life increase the likelihood of developing wheezing illnesses and contribute to asthma development in high-risk children in later life [5,6,7]. Similarly, in nasopharyngeal samples of adults with lower respiratory illnesses, it has been found that 35% of patients had sustained viral RNA levels, whereas 65% of patients had HRV re-infections. The HRV re-infections are significantly associated with chronic obstructive pulmonary disease and asthma, indicating that patients with chronic airway co-morbidities may be predisposed to more frequent rhinovirus re-infections [1]. Additionally, more than half of HRV-infected patients were co-infected with other respiratory viruses, while about two-thirds to half of the patients infected with influenza A virus (IAV) were co-infected with other respiratory viruses [8,9].

Influenza has commonly afflicted humans for many centuries. Symptoms associated with influenza virus infection may present as a mild respiratory disease confined to the upper respiratory tract, and are characterized by fever, sore throat, runny nose, cough, headache, muscle pain, and fatigue. However, influenza may be severe and even culminate in lethal pneumonia, in some cases owing to influenza virus or to secondary bacterial infection of the lower respiratory tract [10]. Pandemic influenza occurs every 10 to 50 years, and is characterized by the introduction of a new IAV strain that is antigenically very different from previously circulating strains. The lack of pre-existing immunity in humans is, therefore, often associated with the increased virulence, severity, and mortality [10]. Interestingly, there is evidence that the interactions between co-circulating and taxonomically different respiratory viruses can influence patterns of infection. Viral interference interactions at the host level are considered important in influencing observed population dynamics. Studies reported that the autumn 2009 epidemic of HRV may have delayed the spread of pandemic H1N1 in several countries in Europe [11,12,13]. The higher rate of HRV infections in 2014 may also have affected the subsequent summer peak of influenza, and even prevented the influenza epidemic in Hong Kong [14]. Asynchronous epidemic peaks of HRV and IAV infections in adult patients were recorded during the 2017 to 2019 winter seasons at Yale—New Haven Hospital, USA [15].

Studies have reported that HRVs and IAVs interact negatively at the population, individual, and cellular levels [16,17]. Given that multiple respiratory viruses co-circulate in the community, and that multiple infections and re-infections occur throughout life [18,19], there is the possibility of cross-protective immunity or compromised immune responses when another viral infection supercedes prolonged HRV infection. Co-infections are generally believed to exert a negative effect on health since co-infected patients display more serious health effects compared to patients with a single infection [20]. Patients with chronic airway co-morbidities are also susceptible to more frequent HRV re-infections [1]. One study evaluated the immune responses and nature of HRV re-infections using mouse-adapted HRV, and found that subsequent heterologous infection following HRV infection induced an intensified, asthma-like phenotype that was dependent on group 2 innate lymphoid cells or ILC2 [21]. In contrast, other studies explored the protective effect against influenza infection following HRV infection. HRV infection of differentiated human airway epithelial cells in vitro at 3 days prior to subsequent IAV infection maintains an interferon (IFN) response that protects against subsequent IAV infection for up to another 3 days [15]. Similarly, HRV infection in human bronchial epithelial cells (hBECs) and human nasal epithelial cells (hNECs) two days prior to a secondary IAV infection can suppress IAV titers more effectively than control-exposed cells [17]. One mouse model also reported that the inoculation of mouse-adapted HRV two days before IAV infection attenuated the disease severity (i.e., clinical signs and bodyweight loss) and mortality; however, HRV was less effective at protecting mice when challenged concomitantly with IAV [22]. These studies imply that the protective effect of HRV is not only associated with an early and controlled inflammatory response, but that the late stage of primary HRV infection is still capable of rendering protection of and driving rapid clearance of IAV. However, it is unclear (a) how the prolonged primary HRV infection modulates successive HRV infection or infection with other more pathogenic respiratory viruses (such as IAV) at the portal of entry of the human airway (i.e., the nose); and (b) whether subsequent activation of immune responses protects more effectively against secondary virus infection or increases the susceptibility of the nasal epithelium. The mechanisms and interference of sequential viral infection, which may affect the pathogenesis and virulence of subsequent viral infections, are poorly understood. It is postulated that the sequence of infections (i.e., preceding infection altering the host response), order of infection of the host, time interval between viral exposures, and route of infection affect the pathogenicity and disease manifestation of the co-infection [23]. It is critical to investigate the protective and aggravating effects of prior exposure to HRV infection, particularly the effect of prolonged primary HRV infection, against secondary virus infections in a systematic and controlled manner in order to elucidate the mechanisms and interactions of dual virus infections in the human airway. Therefore, this study explored the effects of acute secondary virus infections following prolonged primary HRV infection. In our previous study, we found that prolonged HRV infection promptly stimulates antiviral interferons (IFNs) and chemokines during the acute stage of infection. Expression levels of RIG-I and interferon-stimulated genes (ISGs) are sustained together with the detection of persistent expression of viral RNA and viral proteins at the late stage of the 14-day infection period [24]. While other studies have explored the protective effect of initial acute HRV infection on secondary IAV infection [15,22], the susceptibility of hNECs to re-infection with the same HRV serotype and to secondary influenza virus infection after primary prolonged HRV infection has hitherto not been studied in detail. Also poorly understood are the effects of “remnants” of viral proteins and of an HRV-induced sustained antiviral state (following diminished production of infectious HRV) against secondary IAV infection. Therefore, our study examined the protective defense of antiviral state induced during prolonged primary HRV infection against secondary IAV infection, using our in vitro model of hNECs.

## 2. Materials and Methods

### 2.1. Derivation of hNESPCs and In Vitro Differentiation of hNECs

Approval to conduct this study was obtained from the National Healthcare Group Domain-Specific Board of Singapore (DSRB code D/11/228) and the Institutional Review Board of the National University of Singapore (IRB code 13-509). In this study, human nasal epithelial stem/progenitor cells (hNESPCs) were derived from tissue biopsies of 18 subjects who underwent septal plastic surgery at the National University Hospital, Singapore. The medical backgrounds of the donors are summarized in Supplementary Table S1. The subjects were not on any oral or topical corticosteroid medications one month prior to surgery, and had no viral infection at the time of surgery. Briefly, tissue specimens were subjected to overnight digestion by Dispase II (Sigma-Aldrich, St Louis, MO, USA) at 4 °C and 15 min trypsinization at 37 °C to obtain primary hNESPCs. A single-cell suspension was seeded onto mitomycin-C (Sigma-Aldrich)-treated NIH/3T3 feeder layer cells for the expansion of hNESPCs for 3–5 d at 37 °C. Then, 1 × 10^5^ hNESPCs were seeded onto Transwell^®^ inserts 12 mm in diameter with 0.4 µm polyester membrane (Corning, NY, USA) for the air-liquid interface (ALI) culture using PneumaCult™-ALI Medium (Stemcell Technologies, Vancouver, BC, Canada). The differentiation medium was refreshed every 48–72 h, and the mucus secreted in the apical chamber was removed while changing the medium. Fully differentiated hNECs, including beating ciliated cells and mucus-producing goblet cells, were obtained after 21–28 d of ALI culture. 

### 2.2. HRV16 and H3N2 Inoculation into Fully Differentiated hNECs and Viral Plaque Assay

HRV16 strain 11757 (ATCC VR-283) was obtained from the American Type Culture Collection (ATCC, Manassas, VA, USA). HRV16 propagation and infection were performed as described previously [24]. The human IAV Aichi/2/1968 H3N2 strain was purchased from the American Type Culture Collection (ATCC, Manassas, VA, USA) and propagated in egg; then, infection was performed as described previously [25]. Briefly, the virus was thawed on ice and immediately diluted using PneumaCult™-ALI Medium at a MOI of 2.5 for HRV16 and a MOI of 0.01 for H3N2. Afterward, 150 µL of inoculum was added to apical chamber of each Transwell insert in 12-well plates and incubated for 1 h at 33 °C before removal of the inoculum. PneumaCult™-ALI Medium without virus was added to hNECs for mock infection. After 14 days of primary HRV infection, HRV and H3N2 were inoculated on hNECs on Transwell for 1 h at 33 °C. The inoculum was removed and the hNECs were incubated for 24 h before harvest. Viral quantification using a plaque assay was performed as described previously [24,25]. Briefly, 1× dPBS was incubated in the apical chamber during harvest for 10 min at 33 °C to collect the apical secretion. Samples were stored at −80 °C in the freezer until titration by plaque assay. HeLa cells at 85–95% confluence in 24-well plates were incubated with 100 µL of serial dilutions, from 10^−1^–10^−6^ of virus, from infected hNECs at 33 °C for 1 h for HRV. MDCK cells, at 85–95% confluence, were incubated in 24-well plates with 100 µL of serial dilutions, from 10^−1^–10^−6^ of virus, from infected hNECs at 35 °C for 1 h for H3N2. The plates were rocked every 15 min to ensure equal distribution of the virus. The inocula were removed and replaced with 1 mL of Avicel (FMC BioPolymer, Philadelphia, PA, USA) overlay in each well, then incubated at 33 °C or 35 °C for 65–72 h for HRV or H3N2, respectively. The Avicel overlay was removed after the incubation period, and cells were fixed with 4% formaldehyde in 1× PBS for 1 h. Formaldehyde was removed, and cells were washed with 1× PBS. The fixed cells were stained with 1% crystal violet for 15 min and subsequently washed. The plaque-forming units (PFU) were calculated as follows: Number of plaques × dilution factor = number of PFU per 100 µL. 

### 2.3. Cell Viability Assay

Cell viability was assessed using the CyQUANT™ LDH Cytotoxicity Assay (Thermo Fisher Scientific, Scoresby, Australia). Briefly, the hNECs on the transwell membrane were washed in 100 µL of 1× dPBS at 37 °C for 10 min and collected. Next, 50 µL of each sample was added into each well in duplicate, and plates were incubated at room temperature for 0.5 h. Absorbance was measured at 490 nm and 680 nm, and the percentage reduction was expressed as a percentage of total LDH released upon lysis of the hNECs with the lysis buffer provided in the kit. 

### 2.4. RNA Extraction and Quantitative Real-Time PCR for Host Immune Markers and HRV Viral Load

Total cell RNA was extracted from hNECs using a mirVana miRNA isolation kit (Life Technologies, Grand Island, NY, USA), following the manufacturer’s protocol. RNA concentration and purity were measured using NanoDrop2000 UV-Vis Spectrophometer (Thermo Fisher Scientific, Waltham, MA, USA), and 1000 ng of total cellular RNA (containing viral RNA) were used for cDNA synthesis using qScript cDNA SuperMix (Quanta BioSciences, Beverly, MA, USA). SYBR Green qPCR was performed as previously described, and the ribosomal protein L13a (RPL13A) housekeeping gene was used for normalization [24]. All primers used are listed in Supplementary Table S2.

### 2.5. H3N2 RT-PCR Amplification and Quantitative Real-Time PCR for H3N2 Viral Load

A 1-µg sample of total cellular RNA (containing viral RNA), previously extracted using the mirVana miRNA isolation kit, was used for cDNA synthesis with MMLV reverse transcriptase (Promega, Madison, WI, USA). 1 µg of RNA with 1 µL of random primers was heated at 70 °C for 5 min and placed on ice immediately. Reverse transcription (RT) using MMLV reverse transcriptase reaction mix was performed at 37 °C for 1 h. 

Real-time quantitative PCR was carried out using primers specific to influenza virus genes. The thermal cycling conditions were 95 °C for 10 min followed by 40 cycles, each consisting of 95 °C for 10 s, 53 °C for 5 s, 72 °C for 1 min, and a final hold at 65 °C for 1 min. The primer sequences used are shown in Supplementary Table S2.

### 2.6. Western Blotting

The preparation of cell lysates, SDS-PAGE, and Western blot analyses were performed as previously described [24]. Cells were lysed using RIPA buffer (Thermo Fisher Scientific, #89900) containing Halt protease and phosphatase inhibitor cocktail (Thermo Fisher Scientific, #78442). Cell lysates were incubated on ice for 20 min and centrifuged at 13,200 rpm for 10 min at 4 °C. The protein concentrations of the samples were assayed using the Pierce BCA Protein Assay Kit (Thermo Fisher Scientific, #23225). Protein samples (30 μg/sample) were loaded and separated with 10–12% SDS-PAGE and transferred to PVDF membranes. Membranes were blocked with 5% skimmed milk in 1× TBST at room temperature for 1 h, then incubated in the primary antibody solution at 4 °C overnight. The following primary antibodies (and dilutions) were used: HRV VP2 (1:1000; QED Bioscience, #18758), RIG-I (1:1000; Abgent, #AP1900A), MDA5 (1:1000; Enzo Life Sciences, #ALX-210-935), IAV NS1 (1:1000; Thermo Fisher Scientific, #32243), IAV M1 (1:1000; Thermo Fisher Scientific, #32253,), MX1 (1:1000; Cell Signaling Technology, #37849), ISG15 (1:500; Cell Signaling Technology, #2743), IFITM1 (1:20,000; Proteintech, #60074-1-Ig), and GAPDH (1:10,000; Abcam, #ab8245). The membranes were incubated with anti-mouse or anti-rabbit HRP-conjugated secondary antibody in 5% milk solution (in 1× TBST). The signal was detected with Clarity™ Western ECL Substrate (Bio-Rad, #1705060). Semi-quantitative analysis was performed on the Western blot bands, and the intensities of the bands were quantified using ImageJ software.

### 2.7. Statistical Analyses

The qPCR and Western blot results were analyzed using GraphPad Prism 6 software (San Diego, CA, USA). For qPCR, since the gene expression levels (2^−ΔΔCt^) were not normally distributed (Gaussian distribution) due to the variability among the different individually-derived hNECs that were analyzed by GraphPad, the median and interquartile ranges were used in the statistical analysis. The significance level was calculated using one-way ANOVA and the non-parametric, grouped, Dunn’s multiple comparisons test, unless otherwise stated. Data are presented as fold-change, and *p*-values of <0.05 were considered significant.

## 3. Results

### 3.1. Viral Replication Dynamics and Activation of Innate Immune Responses during Secondary H3N2 Infection and HRV-A16 Re-Infection in hNECs

**Figure 1:**
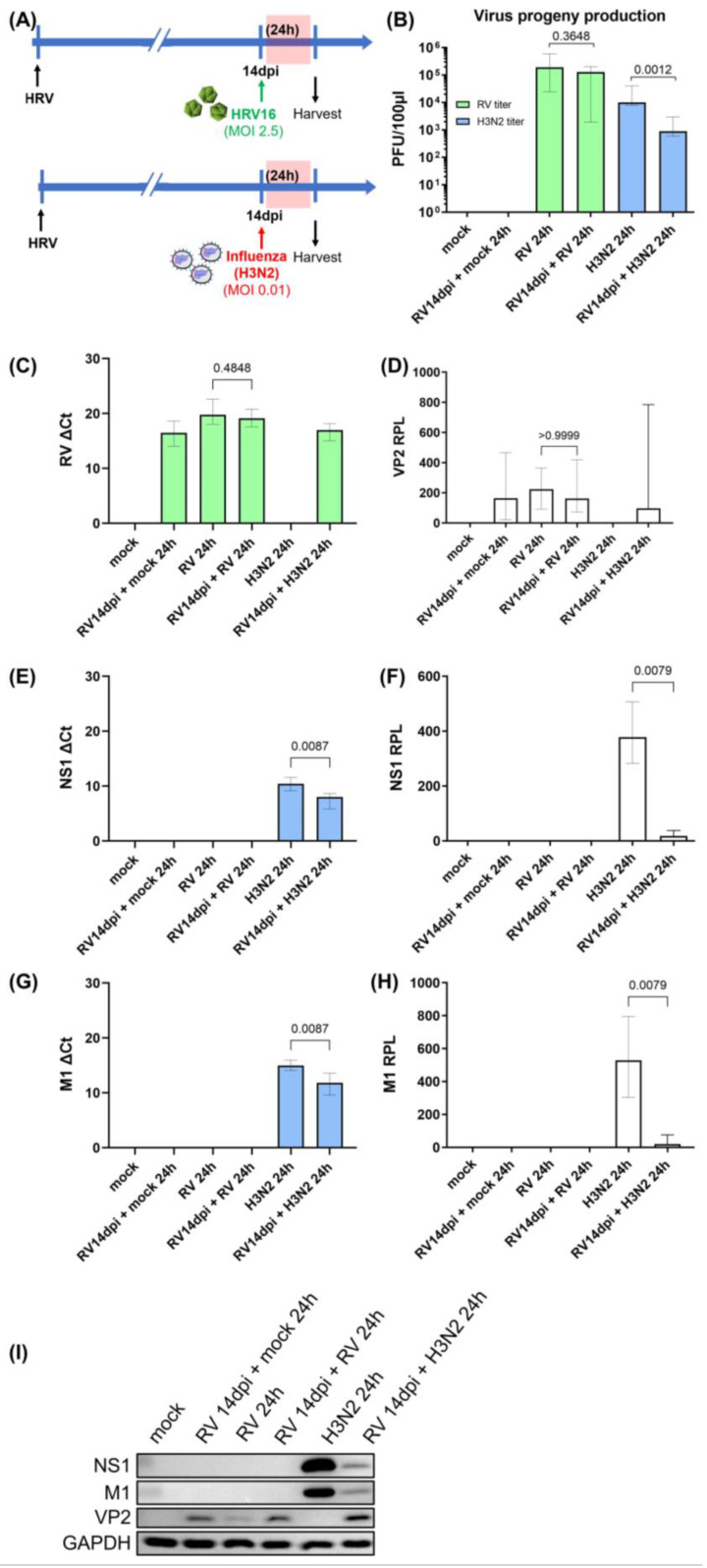
Reduction in virus progeny production and expression of IAV NS1 and M1 proteins of secondary H3N2 infection, but not HRV VP2 of HRV-A16 re-infection in hNECs (following primary prolonged HRV infection). (**A**) Timeline of prolonged primary HRV infection and subsequent secondary H3N2 infection or HRV-A16 re-infection. (**B**) Infectious virus progeny was quantified (PFU per 100 μL) using plaque assay (*n* = 7). There were no significant changes in (**C**) HRV RNA or (**D**) VP2 protein of HRV re-infection as compared to single HRV infection and controls. (**E**–**H**) However, IAV RNAs and relative protein levels (RPL) of NS1 and M1 of secondary H3N2 infection were significantly reduced (*n* = 5). The relevant band intensities were measured using ImageJ software. The corresponding *p*-values are shown. The *p*-values were calculated by comparison with single infection of the respective virus using the non-parametric Mann–Whitney U-test. The data are represented as medians with interquartile values. (**I**) Representative Western blot images are shown.

Our previous study reported that viral RNA and viral protein VP2 were detected 14 days after initial HRV infection. Sustained expression of RIG-I and ISGs was also observed during the prolonged HRV infection model of hNECs. Viral loads of secondary H3N2 infection following various periods of primary HRV infection were examined. By temporal analysis, we observed the greatest reduction in IAV progeny production (of secondary H3N2 infection) after 4 days of primary HRV infection (Supplementary Figure S1). To follow up on our previous study, we focused on the investigation of secondary H3N2 and HRV-A16 re-infection after 14 days of primary HRV infection, which was associated with a sustained antiviral state without active production of infectious HRV particles (Figure 1A). The IAV progeny production of secondary H3N2 infection for 24 h was significantly reduced as compared to single IAV infection (Figure 1B). Congruently with this, the expression levels of IAV NS1, M1 RNAs, and proteins of secondary H3N2 infection were also significantly reduced as compared to single IAV infection (Figure 1E–H). In contrast, there was no significant change in the HRV RNA and VP2 protein expression in HRV-A16 re-infection as compared to single HRV infection (Figure 1C,D). 

**Figure 2:**
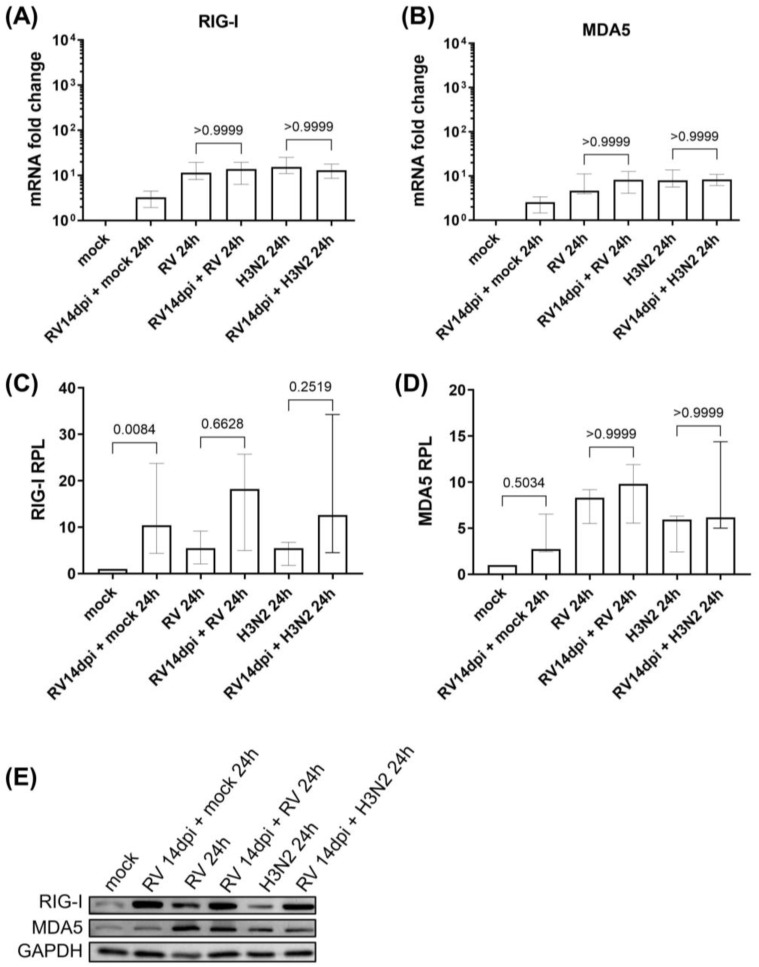
Increased protein expression of RIG-I, but not MDA5, during secondary H3N2 infection and HRV-A16 re-infection of hNECs (following primary prolonged HRV infection). The mRNA expression profiles of (**A**) RIG-I and (**B**) MDA5 in infected and mock control hNECs (*n* = 6). Relative protein levels (RPL) of (**C**) RIG-I and (**D**) MDA5 in infected and mock control hNECs (*n* = 5). The relevant band intensities were measured using ImageJ software. Protein levels were normalized to GAPDH housekeeping protein. (**E**) Representative Western blot images are depicted. The *p*-value was calculated by one-way ANOVA and non-parametric Kruskal–Wallis test. The data are represented as medians with interquartile values.

To analyze the susceptibility of hNECs after prolonged primary HRV infection against subsequent secondary IAV infection and HRV re-infection, we examined the cytoplasmic pathogen sensors during secondary H3N2 infection and HRV-A16 re-infection. There were no significant changes in the mRNA expression of RIG-I and MDA5, nor in MDA5 protein expression, during secondary H3N2 infection or HRV-A16 re-infection. However, the protein expression of RIG-I increased in the subsequent infections as compared to the respective single infections (Figure 2A–E). Similarly to our previous study, we also observed a significant elevation in baseline RIG-I protein expression after 14 days of prolonged HRV infection (even without sequential infection of hNECs). 

Our data also showed no significant alterations in the mRNA expression of antiviral IFNs (namely, IFN-β1 and IFN-λ1) nor in the signature chemokine CXCL10, during the secondary H3N2 infection and the HRV-A16 re-infection as compared to their respective single infections in hNECs (Supplementary Figure S3A–C). 

**Figure 3:**
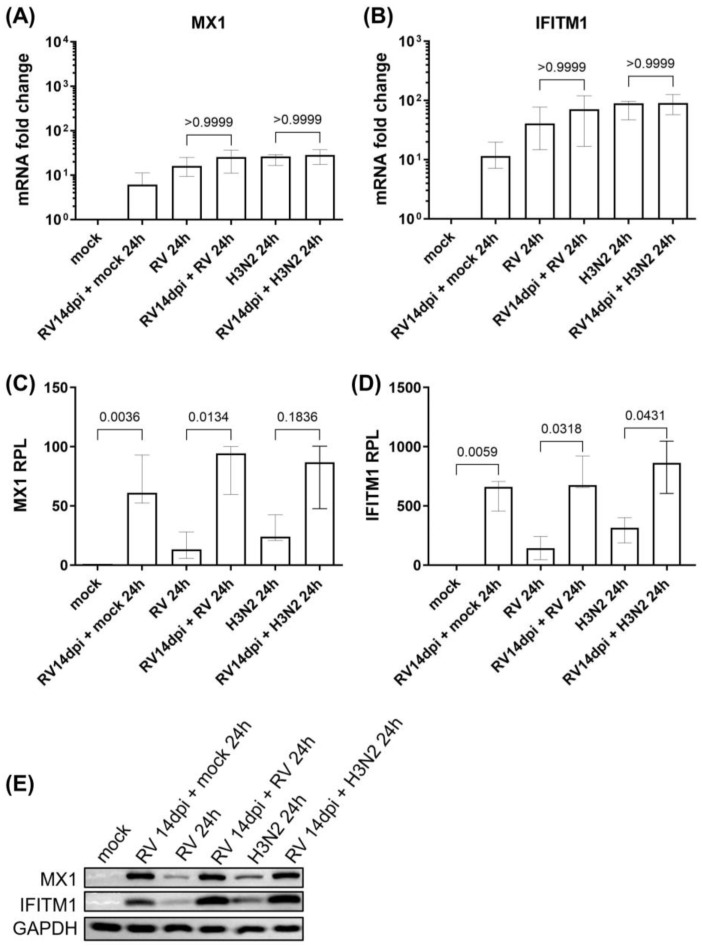
Increased expression of MX1 and IFITM1 proteins during secondary H3N2 infection and HRV-A16 re-infection of hNECs (following primary prolonged HRV infection). The mRNA expression profiles of ISGs (**A**) MX1 and (**B**) IFITM1 in infected and mock control hNECs (*n* = 6). Relative protein levels (RPL) of (**C**) MX1 and (**D**) IFITM1 protein expression in infected and mock control hNECs (*n* = 5). The relevant band intensities were measured using ImageJ software. Protein levels were normalized to GAPDH housekeeping protein. (**E**) Representative Western blot images are shown. The *p*-value was calculated by one-way ANOVA and non-parametric Kruskal–Wallis test. The data are represented as medians with interquartile values.

In our previous study, we found that the gene expression of ISGs was sustained throughout 14 days of HRV infection. Herein, we observed no significant change in the mRNA expression profiles of the antiviral ISGs MX1 and IFITM1 during secondary H3N2 infection nor HRV-A16 re-infection as compared to their respective single infections in hNECs (Figure 3A,B). Interestingly, however, the protein expression levels of MX1 and IFITM1 during secondary H3N2 infection and HRV-A16 re-infection were significantly increased as compared to their respective single infections (Figure 3C–E). We also validated the significant elevation in the baseline protein expression of MX1 and IFITM1 after 14 days of prolonged primary HRV infection (even without sequential infection of hNECs).

### 3.2. Viral Replication Dynamics and Activation of Innate Immune Responses after Longer Rupintrivir Treatment Prior to Secondary H3N2 Infection of hNECs

**Figure 4:**
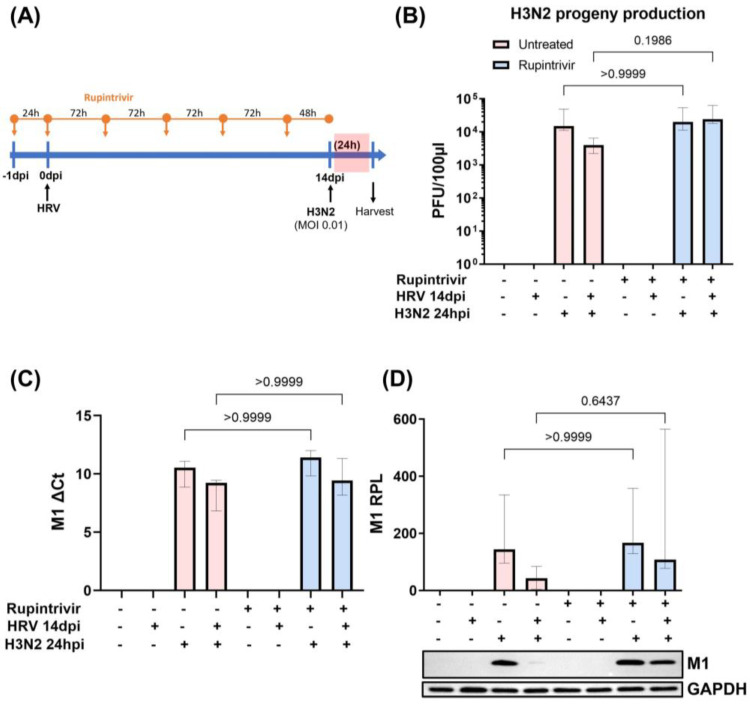
Earlier and longer-duration Rupintrivir-mediated inhibition of HRV 3C protease from prolonged primary HRV infection abolished the reduction in live influenza viral load of secondary H3N2 infection. (**A**) Timeline of early and multi-dose Rupintrivir treatment prior to secondary H3N2 infection of hNECs. (**B**) Infectious virus progeny was quantified (PFU per 100 μL) using virus plaque assay (*n* = 6). (**C**) The mRNA levels (*n* = 4) and (**D**) relative protein levels (RPL) (*n* = 6) of IAV M1 of secondary H3N2 infection, with and without Rupintrivir treatment, in infected and mock control hNECs. Representative Western blot images are shown. The relevant band intensities were measured using ImageJ software. Protein levels were normalized to GAPDH housekeeping protein. The *p*-value was calculated by one-way ANOVA and non-parametric Kruskal–Wallis test. The data are represented as medians with interquartile values.

We examined whether inhibition of the HRV 3C protease from the prolonged primary HRV infection could impact the influenza viral load of secondary H3N2 infection. Thus, Rupintrivir (a HRV 3C protease inhibitor) was added to hNECs one day prior to secondary H3N2 infection (Supplementary Figure S5A). Initially, our data revealed that delayed single-dose Rupintrivir pre-treatment prior to secondary H3N2 infection did not significantly alter the live IAV progeny production during secondary H3N2 infection of hNECs as compared to that without pre-treatment (Supplementary Figure S5B). With single-dose Rupintrivir pre-treatment, IAV M1 protein expression during secondary H3N2 infection of hNECs was still reduced (albeit not statistically significantly) compared to that during the single IAV infection (Supplementary Figure S5C,D). Hence, we performed earlier multi-dose Rupintrivir treatment prior to secondary H3N2 infection (Figure 4A), which led to a reversion of the level of live IAV progeny production of secondary H3N2 infection to that of single IAV infection (Figure 4B). However, the longer-duration Rupintrivir treatment did not revert the IAV RNA nor the IAV protein levels to those observed during single H3N2 infection (Figure 4C,D). 

We validated that earlier and longer-duration treatment with Rupintrivir effectively reduced HRV VP2 protein production, despite only lowering HRV RNA to a relatively smaller extent (Supplementary Figure S6A,B). With a longer duration of Rupintrivir treatment, there was no significant alteration in RIG-I mRNA expression but there was a decreasing trend in RIG-I protein level during secondary H3N2 infection of hNECs as compared to that without treatment (Supplementary Figure S6C,D). Longer-duration Rupintrivir treatment also showed no significant alterations in mRNA expression profiles of antiviral IFNs (IFN-β1 and IFN-λ1), nor in the signature chemokine CXCL10, during secondary H3N2 infection or HRV-A16 re-infection as compared to infection of hNECs without treatment (Supplementary Figure S6E–G). 

**Figure 5:**
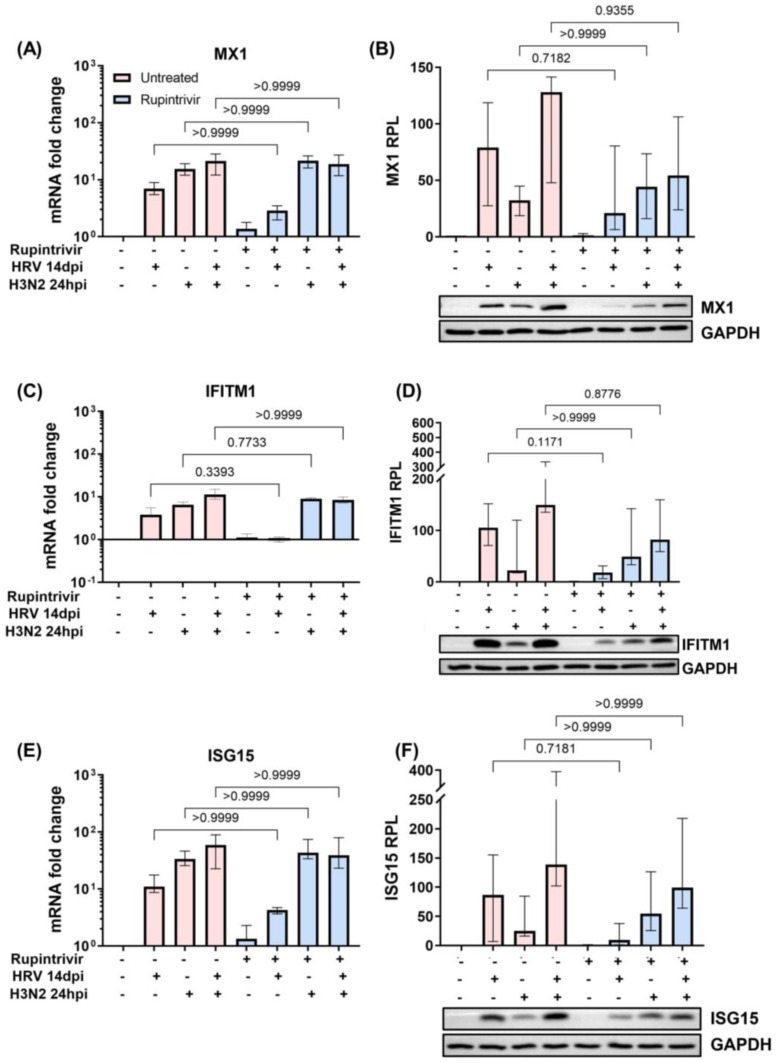
Earlier and longer-duration Rupintrivir inhibition of 3C protease from primary prolonged HRV infection led to decreasing trends in protein levels of ISG proteins MX1, IFITM1, and ISG15 during secondary H3N2 infection. The mRNA fold changes (*n* = 4) and relative protein levels (RPL) (*n* = 6) of (**A**,**B**) MX1, (**C**,**D**) IFITM1, and (**E**,**F**) ISG15 during secondary H3N2 infection, with and without longer-duration Rupintrivir treatment, in mock and infected hNECs. Representative Western blot images are shown. The relevant band intensities were measured using ImageJ software. Protein levels were normalized to the host GAPDH protein. The *p*-value was calculated by one-way ANOVA and non-parametric, Kruskal–Wallis test. The data are represented as medians with interquartile values.

However, secondary H3N2 infection of hNECs with earlier and longer-duration Rupintrivir treatment culminated in decreasingly trending relative protein levels (RPL) of MX1, IFITM1, and ISG15 compared to without treatment, albeit without statistical significance (Figure 5A–F). However, this decreasing trend was not observed when secondary H3N2 infection was subjected to delayed and single-dose Rupintrivir treatment (Supplementary Figure S7A–G).

## 4. Discussion

Our previous study found that prolonged HRV infection induces sustained expression of viral components (HRV RNA and protein) and antiviral host factors (particularly RIG-I and ISGs) until the late stages of infection [24]. However, it is unclear how prolonged primary HRV infection modulates subsequent infection with rhinovirus or other, more pathogenic, respiratory viruses (i.e., influenza A virus) at the nasal portal of entry of the human airway, and whether subsequent immune response activation confers protection for or increases the susceptibility of the nasal epithelium against secondary virus infection. Therefore, this study examined the effects of primary prolonged HRV infection on HRV re-infection and secondary H3N2 infection. 

We first hypothesized that nasal-specific innate immune responses induced and sustained from primary HRV infection would dampen the viral load of secondary H3N2 infection. By temporal analysis, we determined the period of primary HRV infection which confers the greatest protection against secondary H3N2 infection to be HRV infection of hNECs at 4 dpi. The results of our previous study also corroborate this finding [26]. Our previous study on acute HRV infection of hNECs revealed that innate immune responses (signature of IFN-β1, IFN-λ1, CXCL10) peak at 72 to 96 hpi, thus supporting this as the optimal time for protection against secondary H3N2 infection. Interestingly, we also noticed inter-individual variations in the protection against secondary H3N2 infection, which may be attributed to the differential levels of antiviral responses elicited by primary HRV infection. 

Next, to follow up on our previous study on HRV persistence and its sustained antiviral responses, we focused our present study on secondary acute virus infection after 14 days of prolonged HRV infection. Interestingly, our results showed that the IAV progeny production of H3N2 secondary infection was significantly reduced, whereas there was no significant change in the HRV progeny production of HRV re-infection as compared to their respective single infections. The diminished IAV progeny production of secondary H3N2 infection is likely due to the phenomenon by which one virus competitively suppresses the replication of other coinfecting viruses. Superinfection suppression may occur when persistently infected cells withstand a challenge of a heterologous virus. The mechanisms of such processes are diverse and have not been determined in all cases, but some mechanisms described thus far depend on direct interaction of products of the primary infection with the secondary infecting virus [27,28]. Additionally, for non-IFN-mediated viral interference, competition between two viruses exists for the metabolites, replication sites [29], and those host factors that support virus replication [30,31]. One virus modulates the host machinery in its favor, thereby interfering with the replication of other coinfecting viruses. A requirement for common cellular factors of unrelated viruses indicates that heterologous viral interference can also occur [32]. For example, the antiviral state in a previous infection consists of augmented expression of a combination of enzymes which, if activated, shut down cellular translation [33]. The most critical enzymes are protein kinase R (PKR) and 2′-5′ oligoadenylate synthetase (2′-5′OAS). PKR inactivates eukaryotic translation initiation factor 2 alpha (eIF2α) by phosphorylation, thereby shutting down protein synthesis [34]. The 2′-5′OAS synthesizes unique oligonucleotides, which activate RNAse L, thus initiating the destruction of cellular and viral RNA molecules necessary for translation. Indeed, our results revealed significantly reduced expression of IAV proteins (M1 and NS1) of secondary H3N2 infection as compared to single infection. This suggests that the antiviral state induced and sustained by primary prolonged HRV infection may have interfered with the protein synthesis of secondary H3N2 infection, thereby dampening the generation of infectious IAV particles. 

In addition to non-IFN-mediated viral interference, innate viral interference mediated via IFNs may have culminated in the reduced IAV progeny production of secondary H3N2 infection. Upon binding with their cognate receptors, IFNs induce ISGs, many of which activate numerous cell signaling pathways and regulate the activity of numerous innate immune mediators that non-specifically block virus replication [35,36,37,38]. Indeed, we found that the baseline protein expression levels of cytosolic pattern recognition receptor (PRR) RIG-I, as well as ISGs (MX1 and IFITM1), were significantly elevated prior to secondary infection. Secondary infection of H3N2 and re-infection of HRV could induce higher levels of ISGs as compared to single infections, although no significant change was detected in IFN-β1 and IFN-λ1 mRNA levels. This implies that the antiviral state sustained during primary prolonged HRV infection protects the hNECs and inhibits the secondary H3N2 infection by greatly dampening IAV protein synthesis, and, therefore, its infectivity. 

In contrast, despite the persistent antiviral state induced by primary prolonged HRV infection, there was no change in HRV progeny production after re-infection with the same HRV serotype. Our in vitro data yielded a different outcome than other studies which involved adaptive immunity. One study reported that prior infection with different serotypes of HRV was able to reduce the viral load of HRV re-infection in early-life mice [21]. Similarly, HRV re-infections are almost invariably heterotypic, and the acquired immunity to previous HRV exposure determines the clinical severity and duration of subsequent HRV infections [1,39,40]. These findings suggest that there is limited cross-protective immunity between HRV serotypes, but lasting type-specific immunity also exists in animal and human hosts due to viral clearance by adaptive immunity. Therefore, it may be due to the absence of adaptive immunity in our in vitro hNEC model that re-infection by the same HRV serotype was not suppressed. Interestingly, however, the nasal cell-specific antiviral state induced by prolonged primary HRV infection was able to ameliorate the secondary H3N2 infection, but not HRV re-infection, suggesting that HRV re-infection may be resistant to the protective and antiviral mechanisms of ISGs (and other molecules) derived from primary HRV infection. While HRV is associated with asthma exacerbation, the exact mechanisms of HRV-induced asthma exacerbation are largely unknown. The sustained upregulation of certain ISGs has been documented among asthmatic patients at 6 or 7 days after HRV inoculation [41,42,43], suggesting a potential role of HRV-induced ISGs in the pathogenesis and exacerbation of asthma. These studies led to speculations on the unique characteristics of HRV infections—such as their possible inherent ability to activate different pathophysiologic and clinical conditions in predisposed individuals—which confer variable asthma-triggering capacities and responses to asthma exacerbation treatment. Interestingly, our data shed some light on the possibility of HRV being resistant to ISGs. Despite the distinct capability of HRV persistence to induce sustained upregulation of ISGs in the airway epithelium, as reported in previous in vitro [24] and in vivo studies [41,42,43], our study observed that nasal epithelium predisposed to upregulated ISGs levels did not protect against re-infection with the same HRV serotype. The distinct possibility of resistance of HRV to antiviral ISGs warrants further investigations and validations. Moreover, future studies should compare the effects of secondary infection of hNECs with another HRV serotype. It would also be interesting to investigate the impact of primary IAV infection on secondary HRV infection.

RIG-I and MDA5 belong to the group of RIG-I-like receptors (RLRs) and constitute an intracellular virus-sensing system to regulate type I IFN production, which is independent of toll-like receptors (TLR) [44]. RIG-I functions as a virus sensor which senses extracellular and viral nucleic acids released from virus-infected cells of various cell types, including airway epithelial cells. RIG-I mainly senses Sendai virus (a paramyxovirus), vesicular stomatitis virus (a rhabdovirus), and influenza A virus (an orthomyxovirus), while MDA5 mainly detects picornaviruses [45]. This specificity is attributed to the length of double-stranded RNA (dsRNA), RNA structure, and dsRNA modifications that are recognized by RLRs, rather than base composition [46]. RIG-I engagement consists of short dsRNA with and without 5′ppp moiety, as well as higher-order structure in the form of an RNA duplex or panhandles [47,48,49]. On the other hand, MDA5 generally senses high-molecular-weight (HMW) RNA species, but 5′ppp is not required [46,50]. Using HRV-infected hBECs and specific siRNA transfection, one study found that both MDA5 and RIG-I are required for maximum IFN-β activation, while MDA5, but not RIG-I, is required for IFN-λ1 activation [51]. Indeed, our previous study observed a differential upregulation pattern of RLRs in response to HRV replication during active infectious virus production versus detection of viral RNA only at the late stage of prolonged infection without live virus production. MDA5 upregulation peaked during acute HRV infection in response to active production of infectious virus particles, but subsided in tandem with the reduction in infectious virus progeny production. In this study, there was no difference between HRV re-infection and single HRV infection, suggesting that MDA5 may be targeting newly active, infectious virus replication. This type of replication generates specific higher-order RNA structures during the early phases of re-infection and single infection. 

Our previous study showed that sustained HRV RNA replication can still induce persistent RIG-I upregulation at the late stage of infection. This is likely due to the change in type of dsRNA species as the prolonged infection progresses. Given that the HRV genome replicates in an RNA-dependent manner, it is, thus, likely that dsRNA molecules are present during replication in the cytoplasm, and that dsRNA of differing sizes are potentially recognized by both RNA helicases MDA5 and RIG-I [51]. One study reported differential levels of positive- and negative-sense strands of HRV during replication from days 1 to 5, suggesting that there is likely a change in the composition of the RNA products generated during viral replication [52]. However, despite the increased baseline RIG-I expression, along with the greater RIG-I upregulation in HRV-reinfected hNECs versus single HRV infection, we did not observe a reduction in viral progeny production or viral protein expression in HRV-reinfected hNECs. This is likely due to the viral immune evasion mechanisms of HRV. The removal of 5′-triphosphate and covalent attachment of a viral peptide (VPg) to the 5′ end of picornavirus RNA serves as a viral strategy to avoid recognition by RIG-I [53]. This suggests that the remaining dsRNA structures of HRV at the late stage of prolonged HRV infection may serve as ligands for the activation of RIG-I, and, thus, stimulate the antiviral state without impacting HRV re-infection. Thus, the effects of HRV and RIG-I warrant further investigation. For example, agonists of RIG-I may potentially serve as prophylactic treatments against infection by more virulent respiratory virus strains. 

More importantly, our data revealed that the increased baseline expression of RIG-I and ISGs by primary prolonged HRV infection confers greater protection capabilities to nasal cells by dampening IAV progeny and protein production of secondary influenza infection. RIG-I (but not MDA5) detects IAV infection by recognizing the 5′-triphosphorylated panhandle structure of the viral RNA genome [54]. Independent of its IFN signaling function, RIG-I can also act as an antiviral effector protein by binding to incoming IAV nucleoprotein and delaying the first cycle of replication [55]. Therefore, hNECs predisposed to higher baseline RIG-I expression exhibit reduced IAV protein expression of secondary influenza infection (particularly NS1) in terms of reduced efficiency of IAV immune evasion and augmented host innate immune responses in the nasal epithelium. One distinct immune evasion mechanism of IAV is the inhibition of RIG-I signaling via direct interaction of the viral NS1 protein with RIG-I. The influenza virus inhibits RIG-I-mediated activation of the IFN-β promoter by interfering with the downstream host factors (e.g., inhibition of nuclear translocation of IRF-3) and forming cellular complexes with IPS-1 and RIG-I [56,57]. Furthermore, NS1 also modulates the antiviral factors by binding cellular DNA and blocking the transcription of antiviral genes [58]. RIG-I activation was also shown to be crucial for the protection and rescue of mice infected with influenza at lethal dosages [59]. In addition to the increased baseline RIG-I expression, the enhanced baseline expression of ISGs prior to secondary IAV infection also allows host innate immune defenses to interfere with IAV replication in a timely manner. One of the ISGs, the MX1 gene product, is located in the cytoplasm of human cells and hinders the secondary steps of viral transcription and replication by retaining incoming viral genomes in the cytoplasm—this blocks the early viral transcription step prior to viral genome replication for new IAV progeny, irrespective of the subcellular site of replication [60,61,62]. Other distinct ISGs, including the family of IFN-induced transmembrane proteins (IFITMs), are designated as cellular antiviral factors that block early viral entry by modifying cellular membrane properties [63,64]. For example, IFITM3 restricts the release of viral contents into the cytoplasm prior to membrane hemi-fusion by blocking fusion pore formation, and alters membrane properties such as curvature and fluidity [65]. Overall, our study revealed that the increased baseline expression of RIG-I and ISGs from primary prolonged HRV infection provided the hNECs with heightened pathogen sensor activity and antiviral states, thereby conferring protection against secondary IAV infection in the nasal epithelium. This effect of hNECs predisposed to elevated RIG-I and ISG expression, which leads to greater and more rapid viral clearance of secondary influenza infection, warrants further investigation.

Our previous study documented sustained expression of HRV protein VP2 at 14 days after initial infection, even in the absence of infectious HRV particle production. We speculate that there is sustained production of other HRV proteins, particularly the 3C protease, that may contribute to the constant triggering of an antiviral state (i.e., RIG-I and ISGs) during prolonged HRV infection, and, thus, may interfere with IAV replication of secondary influenza infection. Since the picornavirus genome contains one open reading frame, which encodes a single polyprotein, cleavage by 3C protease is a key process for the release of mature and functional proteins from the polyprotein [66]. This is critical for protein-primed RNA synthesis initiation and the switch from viral translation to viral replication [67,68,69]. Interestingly, 3C protease is also responsible for immune evasion in terms of rapid shut-off of transcription, rapid inhibition of protein synthesis initiation, and inhibition of nucleocytoplasmic transport by the nuclear pore complex in host cells [70,71,72]. Therefore, we investigated the potential effect of 3C protease in sustaining the antiviral state derived from primary prolonged HRV infection on secondary IAV infection via single-dose Rupintrivir pre-treatment one day prior to secondary H3N2 infection. Rupintrivir is a viral 3C protease inhibitor that forms a covalent bond with the active site cysteine on the viral protease [73]. Our data indicated that the delayed inhibition of 3C protease one day prior to secondary H3N2 infection did not alter the IAV load compared to without pre-treatment. However, one likely explanation is that the delayed and short-term inhibition of 3C protease after the establishment of the network cascade of ISGs did not have a sufficient effect on the RIG-I-IRF3-ISGs pathway due to the positive feedback loop of ISGs [74,75]. IFNs are known to induce ISG expression through phosphorylation of signal transducers and activators of transcription (STAT) STAT1 and STAT2. Positive regulatory mechanisms then augment the IFN signaling cascade to maintain or amplify the expression of ISGs or IFNs [76]. The cascade components involved in amplifying IFN signals include STAT1 and IFN regulatory factor 9 (IRF9), which associate with STAT2 to form the ISGF3 transcription factor that binds to ISG promoter elements (interferon-stimulated response element, or ISRE) of over 300 ISGs. Studies have shown the high complexity of IFN signaling, and the evidence supports the notion that ISG expression patterns are globally sustained in response to IFN. This sustained response relies on prolonged expression of the ISGF3 and GAF components STAT1, STAT2, IRF9, and IRF1 as part of a positive feedback loop, thereby resulting in enhanced viral resistance [77,78,79]. In addition to promoting the secretion of cytokines and chemokines, positive feedback mechanisms can lock cells into an autocrine signaling loop that sustains IFN signal transduction [80]. Moreover, IFN also activates the transcription of RIG-I, thus contributing to the positive feedback loop and amplifying antiviral signals [81]. Therefore, the delayed and short-term inhibition of HRV 3C protease may be insufficient (a) to alter the sustained antiviral state from prolonged primary HRV infection and (b) to alter the ISG signaling against secondary IAV infection.

Interestingly, however, earlier and longer-lasting inhibition of 3C protease by multiple doses of Rupintrivir could partially abolish the reduction of the IAV M1 protein and live IAV progeny production of secondary H3N2 infection which is induced by prolonged primary HRV infection. Prolonged Rupintrivir treatment thus contributed to the diminished antiviral state stimulated by primary HRV infection. This suggests that the increased expression of antiviral factors arising from prolonged primary HRV infection may be attributed in part to HRV 3C protease activity [82]. This also implies that persistent infection of a mild respiratory virus such as HRV may confer a protective effect against a more virulent secondary virus infection such as IAV. Such a phenomenon may be congruent with a study indicating that HRV infections peak each autumn and spring, whereas IAV peaks each winter between the HRV peaks [15]. Our study suggests that the interference of viruses in sequential seasonality may be partially explained by the antiviral state activated by prior HRV infection, which then ameliorates susceptibility to IAV infection efficiency and confers differential protection against secondary IAV infection, with some inter-individual variation [26]. One limitation of our study is that we tested only one strain each of HRV and IAV. Future experiments should be repeated using different HRV and IAV strains and isolates to validate the reported findings and mechanisms.

It is noteworthy that another study reported that SARS-CoV-2 replication was also impaired by primary HRV and IAV infections in upper respiratory tract cells, due to IFN induction from prior infections [83]. In view of the important interactions of immune cells with hNECs during sequential infections, future studies should also investigate co-cultures of infected hNECs with immune cells such as peripheral blood mononuclear cells [84].

## 5. Conclusions

In conclusion, our study revealed that primary prolonged HRV infection diminished the viral load of secondary IAV infection, but not re-infection by HRV of the same serotype. The elevated baseline expression of pathogen sensor RIG-I and ISGs from primary prolonged HRV infection likely interferes with IAV protein synthesis of secondary H3N2 infection, thereby suppressing the generation of infectious virus particles. Delayed single-dose Rupintrivir inhibition of HRV 3C protease derived from primary infection did not alter the IAV load of secondary H3N2 infection. However, earlier and longer-lasting Rupintrivir treatment partially abolished the reduction in active IAV progeny and protein production of secondary H3N2 infection by dampening the sustained antiviral state stimulated by primary HRV infection. Overall, our study highlights that the antiviral state induced from the non-infectious phase of primary prolonged HRV infection can confer protection against secondary IAV infection in the in vitro infection model of hNECs, and that this protection exhibits inter-individual variation. Future in vivo investigations and clinical studies are warranted to study the effects of RIG-I and ISGs on the interference of viral replication of infection with IAV, as well as other respiratory viruses secondary to primary HRV infection. 

## Data Availability

The data presented in this study are available in the Article or Supplementary Materials.

## Supplementary Materials

The following supporting information can be downloaded at: https://www.mdpi.com/article/10.3390/cells12081152/s1, Table S1: Information on patient donors of hNECs. Table S2: Sequences of primers for RT-qPCR using SYBR green. Figure S1: Significant reduction in live influenza virus progeny production of secondary H3N2 infection after primary HRV infection for 1, 4, 8 and 12 days. Figure S2: Negligible cell death was detected during secondary H3N2 infection and HRV-A16 re-infection of hNECs. Figure S3: Profiles of mRNA expression of Type I and Type III interferons (IFNs) and signature chemokine CXCL10 during secondary H3N2 infection and HRV-A16 re-infection of hNECs. Figure S4: Optimization of Rupintrivir concentration for the inhibition of viral 3C protease during HRV infection. Figure S5: Effects of delayed single-dose Rupintrivir inhibition of HRV 3C protease from prolonged primary HRV infection on the live influenza viral load and M1 protein of secondary H3N2 infection. Figure S6: Earlier and longer Rupintrivir inhibition of 3C protease from primary prolonged HRV infection did not significantly affect HRV RNA levels, RIG-I, IFNs, and CXCL10 during secondary H3N2 infection. Figure S7: Delayed and single-dose Rupintrivir inhibition of 3C protease from primary prolonged HRV infection did not significantly affect expression of ISGs during secondary H3N2 infection.
