# Supplementary material for: Prolonged Primary Rhinovirus Infection of Human Nasal Epithelial Cells Diminishes the Viral Load of Secondary Influenza H3N2 Infection via the Antiviral State Mediated by RIG-I and Interferon-Stimulated Genes
Source: Cells. 2023 Apr 13;12(8):1152. doi: 10.3390/cells12081152 (PMC10137142; doi:10.3390/cells12081152)
Supplement: Supplementary file 1 [file cells-12-01152-s001.zip › cells-2035610-supplementary.pdf]

Supplementary Table S1. Information on patient donors of hNECs.

Code	Race	Age	Gender	Allergy*	Asthma#	Smoker
D1	Others	28	M	No	No	No
D2	Others	32	M	No	No	No
D3	Chinese	24	M	No	Yes	No
D4	Chinese	26	M	No	No	No
D5	Others	29	M	No	No	No
D6	Chinese	54	F	No	No	No
D7	Indian	41	M	No	No	No
D8	Chinese	44	F	Yes	No	No
D9	Chinese	31	M	No	No	No
D10	Malay	24	M	Yes	No	Yes
D11	Chinese	35	M	No	No	Yes
D12	Indian	21	M	Yes	No	No
D13	Indian	59	F	No	No	No
D14	Chinese	24	M	No	No	No
D15	Chinese	35	M	No	No	No
D16	Chinese	46	M	No	No	No
D17	Others	54	M	No	No	No
D18	Chinese	51	F	No	No	No

F, female; M, male.* Diagnosis of allergic rhinitis was based on the concordance between a typical history of allergic symptoms and skin prick testing using a local panel of common allergens (e.g. *Dermatophagoides pteronyssinus*, *Dermatophagoides farinae*, *Blomia tropicalis*, *Aspergillus*, *Acacia*, *Cladosporium herbarium*, common ragweed, Bermuda grass, dog dander, cat dander, German cockroach) by the otolaryngologist.# Diagnosis or history of asthma was based on medical records kept at the National University Hospital, Singapore. Patient was recorded to have childhood asthma, and did not receive specific treatment for asthma at least one year prior to surgery.

Supplementary Table S2. Sequences of primers for RT-qPCR using SYBR green.

	Gene symbol	Forward (F) and reverse (R) primer sequences (5′-3′)
5′ untranslated region (5′ UTR) of HRV16	*RV-S1*	F: GCACTTCTGTTTCCCC<br>R: CGGACACCCAAAGTAG
NS1 of H3N2	*NS1-M13*	F: TGTAAAACGACGGCCAGTAGC AAAAGCAGGGTGACAAAGACA<br>R: CAGGAAACAGCTATGACCAGTA GAAACAAGGGTGTTTTTTAT
M1 of H3N2	*M1-M2*	F: CATCCTGTTGTATATGAGGCCCAT<br>R: GGACTGCAGCGTTAGACGCTT
Pathogen sensors	*RIG-I (DDX58)*	F: GGTATAGAGTTACAGGCATTTC<br>R: TTGTTTACTAGTGTTGTGGC
	MDA5 (IFIH1)	F: GATTAAGTGGTGATACCCAAC<br>R: GTCTGACAATTGAACACCAG
Type I interferon	*IFN-β*	F: ATTCTAACTGCAACCTTTCG<br>R: GTTGTAGCTCATGGAAAGAG
Type III interferon	*IFN-λ1 (IL29)*	F: CAGGTTCAAATCTCTGTCAC<br>R: AACTCCAGTTTTTCAGCTTG
Chemokines	*IP-10 (CXCL10)*	F: AAAGCAGTTAGCAAGGAAAG<br>R: TCATTGGTCACCTTTTAGTG
Interferon-stimulated genes (ISGs)	*IFITM1*	F: CTACTCCGTGAAGTCTAGG<br>R: ATGAGGATGCCCAGAATC
	MX1	F: CAGGCTTTGTGAATTACAGG<br>R: TCTTCAATTTTGGACTTGGC
	ISG15	F: AGATCACCCAGAAGATCG<br>R: TGTTATTCCTCACCAGGATG
Housekeeping gene	*RPL13A*	F: GTCTGAAGCCTACAAGAAAG<br>R: TGTCAATTTTCTTCTCCACG

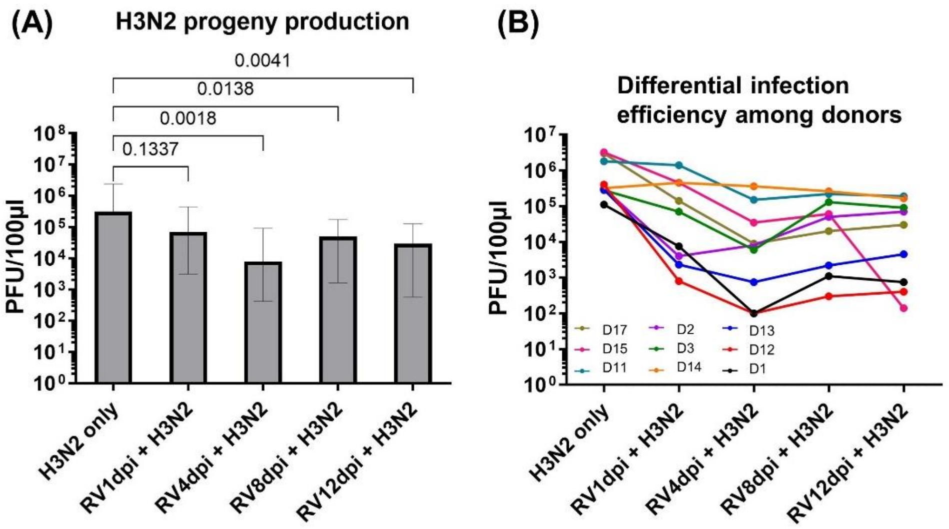

Supplementary Figure S1. Significant reduction in live influenza virus progeny production of secondary H3N2 infection after primary HRV infection for 1, 4, 8 and 12 days. (A) Secondary H3N2 infection was initiated after varying periods of primary HRV infection. Infectious IAV progeny was quantified (PFU per 100 µL) using virus plaque assay (n = 9). (B) Inter-individual differences were observed between hNECs from different donors, e.g. for 4 days of primary HRV infection, donors D1 and D12 exhibited the greatest reduction (3 logs) in viral load of secondary H3N2 infection, whereas donor D14 displayed no change in IAV load (n = 9). The p-value was calculated by one-way ANOVA, non-parametric, Kruskal-Wallis test. The data are represented as medians with interquartile values.

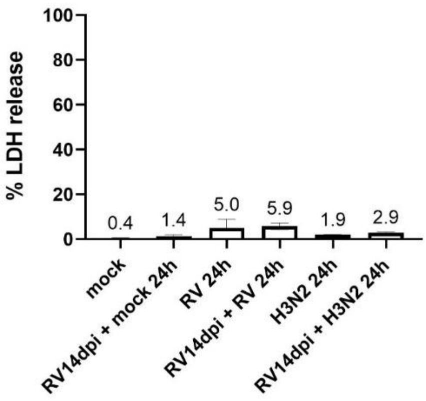

Supplementary Figure S2. Negligible cell death was detected during secondary H3N2 infection and HRV-A16 re-infection of hNECs. The y-axis shows the percentage of LDH released from the apical surface of hNECs with secondary H3N2 infection and HRV-A16 re-infection (n = 3). The p-value was calculated by one-way ANOVA, non-parametric, Kruskal-Wallis test. The data are represented as medians with interquartile values.

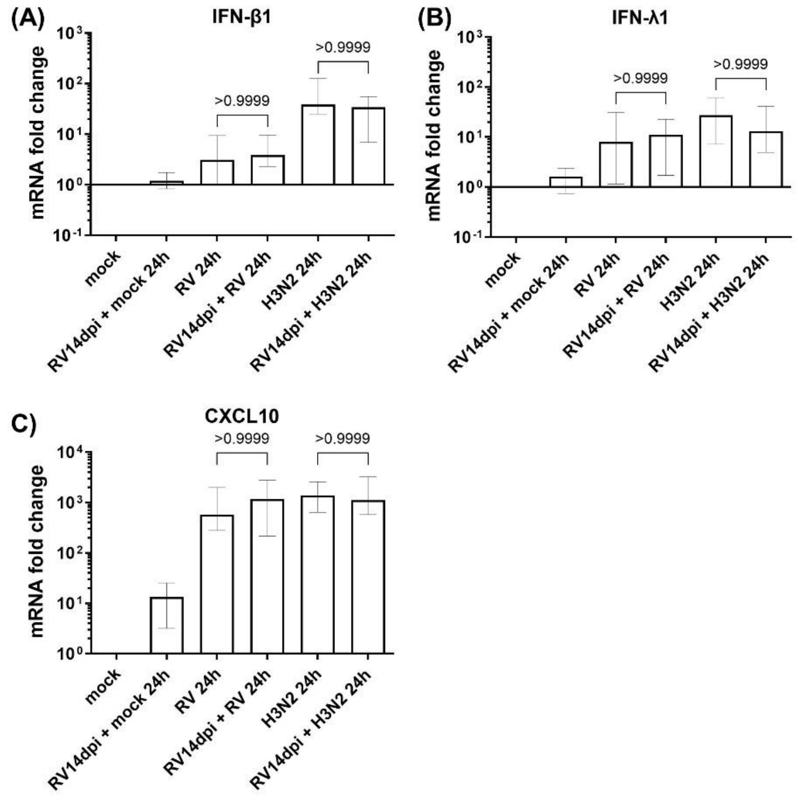

Supplementary Figure S3. Profiles of mRNA expression of Type I and Type III interferons (IFNs) and signature chemokine CXCL10 during secondary H3N2 infection and HRV-A16 re-infection of hNECs. The mRNA expression profiles of (**A**) IFN-β1, (**B**) IFN-λ1, and (**C**) CXCL10 in infected and mock control hNECs (*n* = 6). The *p*-value was calculated by one-way ANOVA, non-parametric, Kruskal-Wallis test. The data are represented as medians with interquartile values.

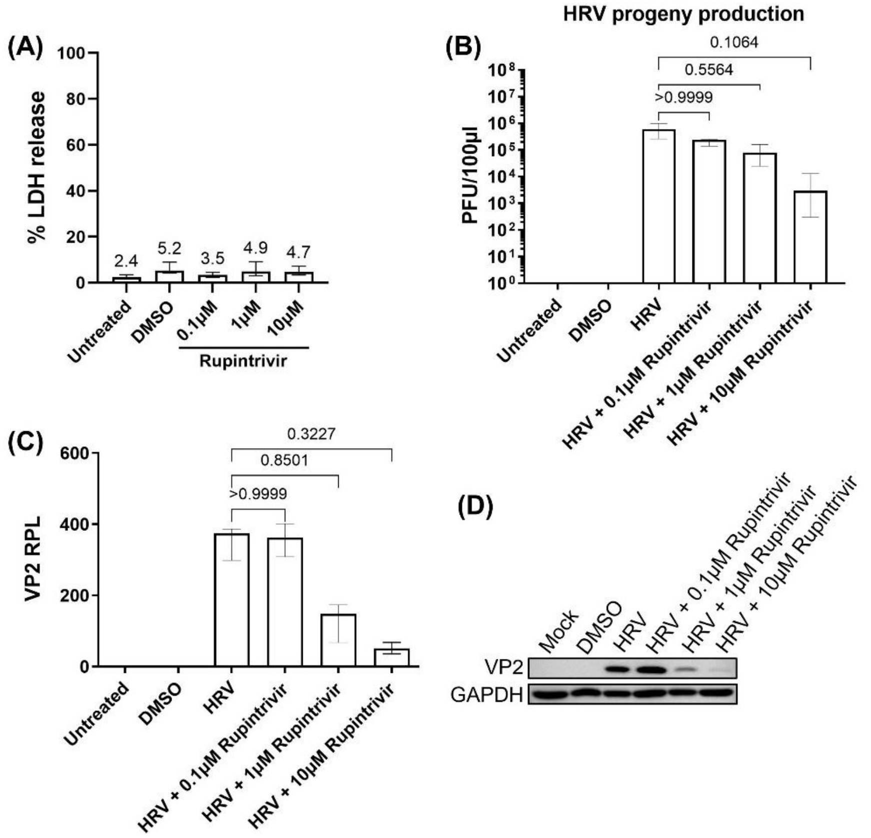

Supplementary Figure S4. Optimization of Rupintrivir concentration for the inhibition of viral 3C protease during HRV infection. (**A**) Percentage of LDH released from apical surface of hNECs with Rupintrivir treatment at 0.1 µM, 1 µM, and 10 µM ($n = 3$). (**B**) Infectious HRV progeny was quantified (PFU per 100 µL) using virus plaque assay ($n = 3$). (**C**) Relative protein level (RPL) of HRV protein VP2 at 24 hpi of HRV-infected hNECs with Rupintrivir treatment at 0.1 µM, 1 µM, and 10 µM ($n = 3$). The relevant band intensities were measured using ImageJ software. HRV VP2 protein levels were normalized to host GAPDH housekeeping protein. (**D**) Representative Western blot images are shown. The *p*-value was calculated by one-way ANOVA, non-parametric, Kruskal-Wallis test. The data are represented as medians with interquartile values.

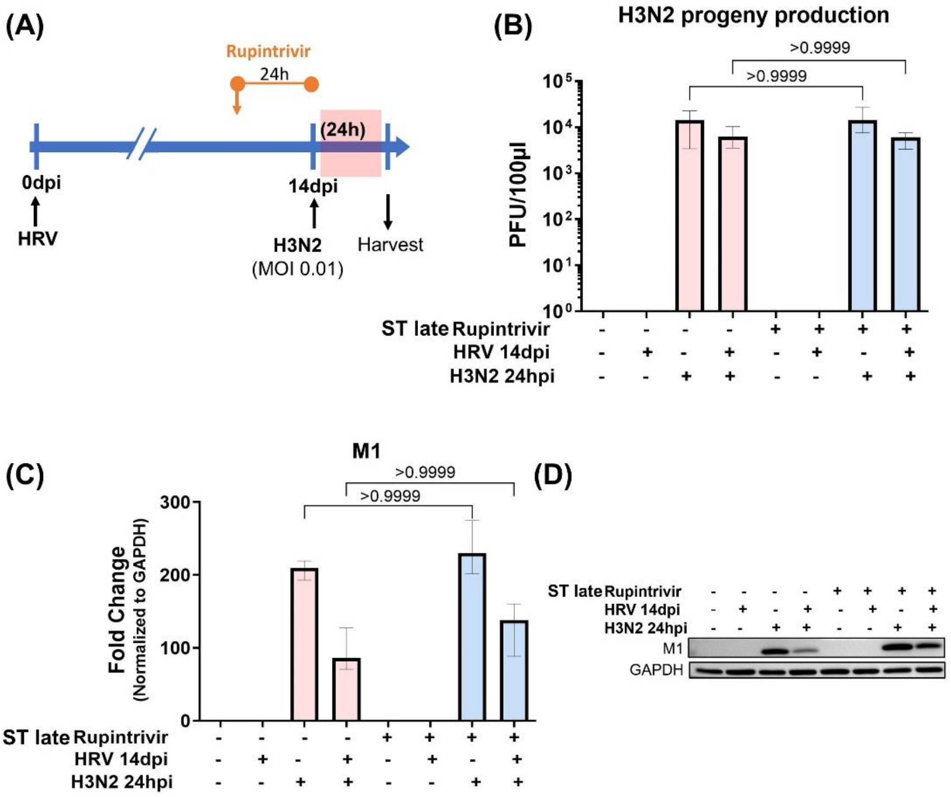

Supplementary Figure S5. Effects of delayed single-dose Rupintrivir inhibition of HRV 3C protease from prolonged primary HRV infection on the live influenza viral load and M1 protein of secondary H3N2 infection. (**A**) Timeline of delayed single-dose Rupintrivir treatment one day prior to secondary H3N2 infection of hNECs. (**B**) Infectious IAV progeny was quantified (PFU per 100 µL) using virus plaque assay (n = 3). (**C**) Western blot analysis of IAV protein M1 of secondary H3N2 infection with and without Rupintrivir pre-treatment in infected and mock control hNECs (n = 4). The relevant band intensities were measured using ImageJ software. Protein levels were normalized to host GAPDH protein. (**D**) Representative Western blot images are shown. The p-value was calculated by one-way ANOVA, non-parametric, Kruskal-Wallis test. The data are represented as medians with interquartile values.

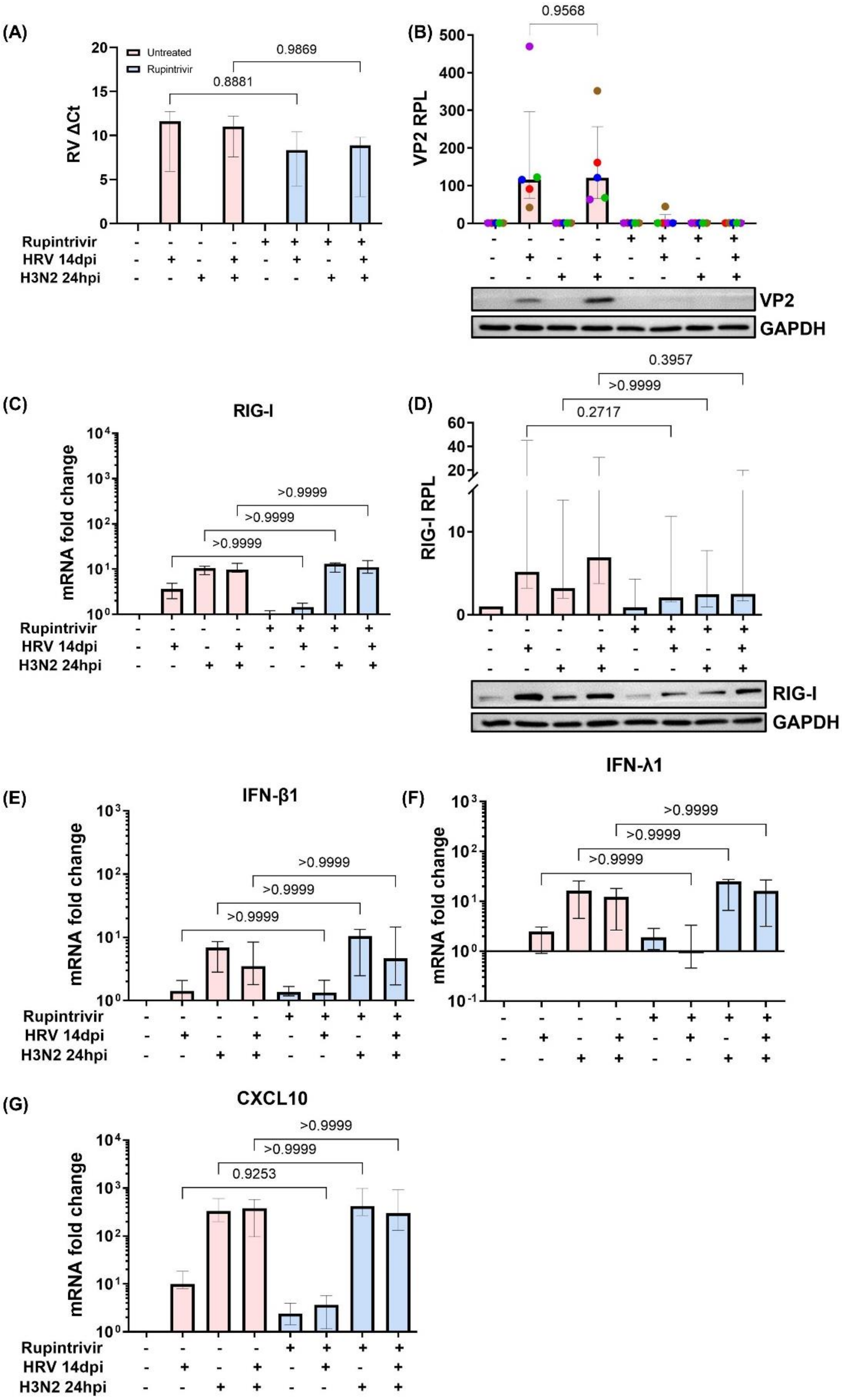

Supplementary Figure S6. Earlier and longer Rupintrivir inhibition of 3C protease from primary prolonged HRV infection did not significantly affect HRV RNA levels, RIG-I, IFNs, and CXCL10 during secondary H3N2 infection. (**A**) There were no significant differences in HRV RNA levels after secondary H3N2 infection with or without longer Rupintrivir treatment (*n* = 4). (**B**) However, HRV VP2 protein expression was inhibited after longer Rupintrivir treatment (*n* = 5).

Inter-individual variations in relative protein levels (RPL) of VP2 were observed for HRV infections without Rupintrivir treatment. (**C–G**) There were no significant changes in mRNA levels of RIG-I, IFN-β1, IFN-λ1 and CXCL10 (n = 4), and relative protein level of RIG-I (n = 6) during secondary H3N2 infection with and without Rupintrivir treatment in mock and infected hNECs. The relevant band intensities were measured using ImageJ software. Protein levels were normalized to host GAPDH protein. Representative Western blot images are shown. The p-value was calculated by one-way ANOVA, non-parametric, Kruskal-Wallis test. The data are represented as medians with interquartile values.

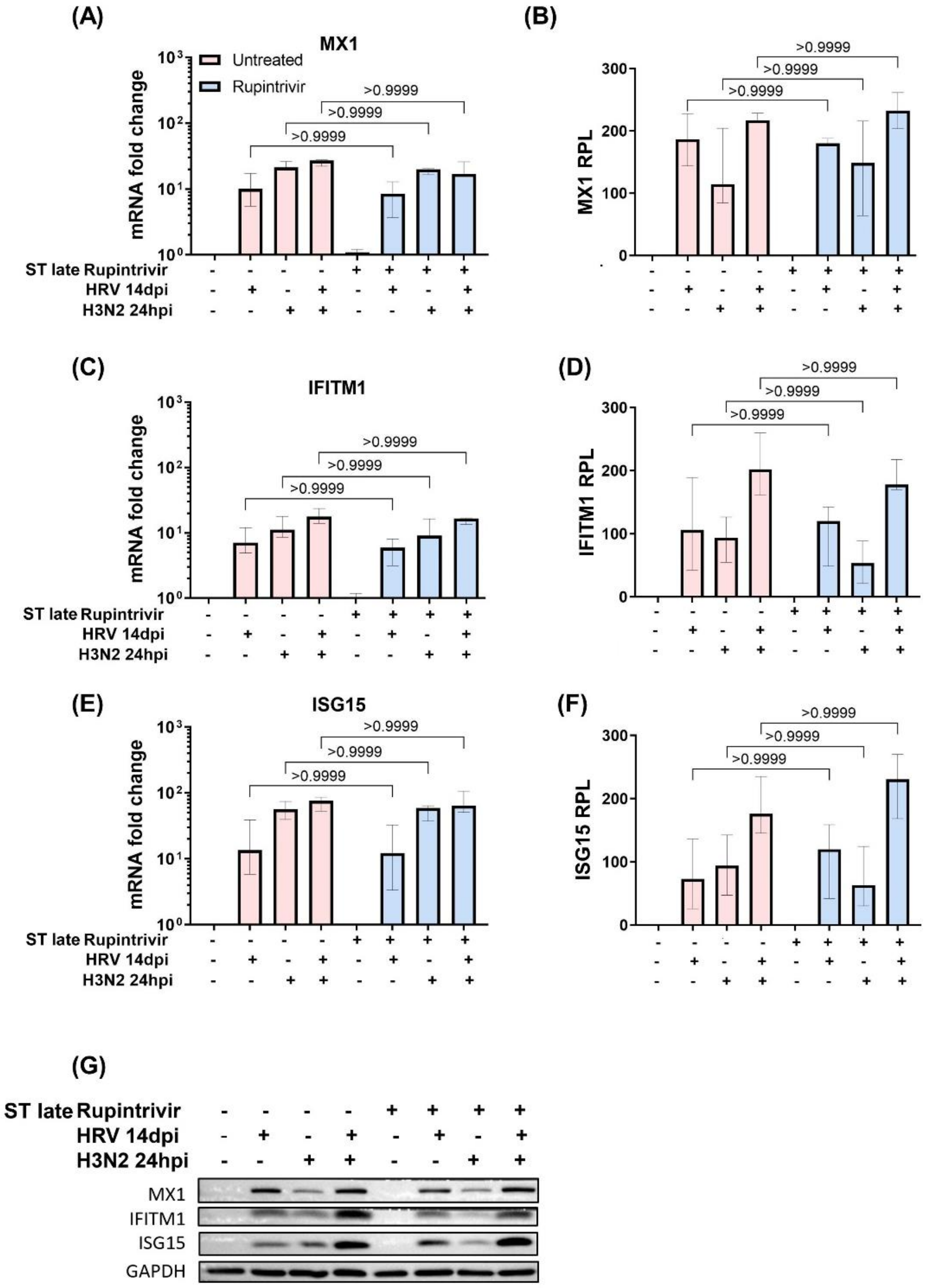

Supplementary Figure S7. Delayed and single-dose Rupintrivir inhibition of 3C protease from primary prolonged HRV infection did not significantly affect expression of ISGs during secondary H3N2 infection. The mRNA fold changes of (**A**) MX1, (**C**) IFITM1 and (**E**) ISG15 during secondary H3N2 infection with and without Rupintrivir treatment in mock and infected hNECs (*n* = 3). The relative protein levels (RPL) of (**B**) MX1, (**D**) IFITM1 and (**F**) ISG15 protein expression

during secondary H3N2 infection with and without Rupintrivir treatment in mock and infected hNECs (n = 4). The relevant band intensities were measured using ImageJ software. Protein levels were normalized to host GAPDH protein. (**G**) Representative Western blot images are shown. The p-value was calculated by one-way ANOVA, non-parametric, Kruskal-Wallis test. The data are represented as medians with interquartile values.
